# Association of Four Genetic Polymorphisms of *AGER* and Its Circulating Forms with Coronary Artery Disease: A Meta-Analysis

**DOI:** 10.1371/journal.pone.0070834

**Published:** 2013-07-24

**Authors:** Feng Peng, Dan Hu, Nan Jia, Xiaobo Li, Yuqiong Li, Shaoli Chu, Dingliang Zhu, Weifeng Shen, Jinxiu Lin, Wenquan Niu

**Affiliations:** 1 Department of Cardiology, the First Affiliated Hospital of Fujian Medical University, Fuzhou, China; 2 Department of Pathology, Fujian Provincial Tumor Hospital, Teaching Hospital of Fujian Medical University, Fuzhou, China; 3 Department of Hypertension, Ruijin Hospital, Shanghai Jiao Tong University School of Medicine, Shanghai, China; 4 Shanghai Institute of Hypertension, Shanghai, China; 5 State Key Laboratory of Medical Genomics, Ruijin Hospital, Shanghai Jiao Tong University School of Medicine, Shanghai, China; 6 Department of Cardiology, Ruijin Hospital, Shanghai Jiao Tong University School of Medicine, Shanghai, China; Baker IDI Heart and Diabetes Institute, Australia

## Abstract

**Background:**

Considerable efforts have been devoted to evaluating the association of the receptor for advanced glycation end-products (gene AGER and protein: RAGE) genetic variants to coronary artery disease (CAD); the results, however, are often irreproducible. To generate more information, we sought to explore four common polymorphisms of *AGER* and its circulating forms associated with the risk of CAD via a meta-analysis.

**Methodology/Principal Findings:**

Articles were identified by searching PubMed, EMBASE, Wanfang and CNKI databases before March 2013. Qualified articles had case-control designs and investigated AGER four polymorphisms (T-429C, T-374A, Gly82Ser, G1704A) or circulating soluble RAGE (sRAGE) or endogenous secretory RAGE (esRAGE) levels associated with CAD. Twenty-seven articles involving 39 independent groups fulfilled the predefined criteria. Overall, no significance was observed for all examined polymorphisms under allelic and dominant models. When restricting groups to CAD patients with diabetes mellitus or renal disease, deviations of risk estimates from the unity were stronger than overall estimates for all polymorphisms except for G1704A due to limited available studies. For example, under dominant model, having -429C allele increased the odds of developing CAD in diabetic patients by 1.22-fold (95% confidence interval (95% CI) 0.99–1.51; P = 0.06; *I* ^2^ = 6.7%) compared with that of overall estimate of 1.15-fold (95% CI: 0.97–1.36; P = 0.111; *I* ^2^ = 18.0%). Circulating sRAGE levels were non-significantly lower in CAD patients than in controls, whereas this reduction was totally and significantly reversed in CAD patients with diabetes mellitus (weighted mean difference: 185.71 pg/ml; 95% CI: 106.82 to 264.61 pg/ml). Circulating esRAGE levels were remarkably lower in CAD patients, as well as in subgroups with or without diabetes mellitus and without renal disease.

**Conclusions:**

Our findings demonstrated that association of *AGER* genetic polymorphisms with CAD was potentiated in patients with diabetes mellitus or renal disease. Practically, circulating esRAGE might be a powerful negative predictor for the development of CAD.

## Introduction

Coronary artery disease (CAD) is a leading cause of morbidity and mortality worldwide. Family studies suggest a strong genetic background: men with 2 or more affected parents or siblings relative to men without family history have a 3.4-fold increased risk of developing myocardial infarction [1]. One of the potential candidate genes that account for an inherited predisposition to CAD is the receptor for advanced glycation end-products (gene: *AGER* and protein: RAGE), which is a multiligand receptor, belonging to the immunoglobulin superfamily of cell surface molecules. The activation of *AGER* can evoke a wide range of signaling pathways that trigger inflammation, atherogenesis and vasoconstriction leading to coronary dysfunction, atherosclerosis and thrombosis [2]. Moreover, circulating soluble RAGE (sRAGE) levels, which were in dose-dependent association with angiographic observations, were observed to be significantly lower in angiographically-confirmed CAD patients than in healthy controls [3], [4]. By contrast, circulating sRAGE levels were significantly higher in patients with acute myocardial infarction, independent of the presence of diabetes mellitus [5]. Therefore association between circulating sRAGE and CAD must be confirmed in larger studies.

The gene encoding *AGER* is mapped on chromosome 6p21.3 and spans 3.27 kb with 11 exons. The genomic sequence of *AGER* gene is highly polymorphic with many alleles that exhibit different functional properties and heterogeneous distributions across populations [6]–[8]. Considerable efforts have been devoted to evaluating the contributory role of *AGER* genetic defects in the development of CAD; the results, however, are not often reproducible. As a caveat, failure to replicate might be attributable to the ethnicity-specific genetic profiles, the individual underpowered studies, and the lack of consideration for confounders. To generate more information, we sought to assess the association of four common polymorphisms (T-429C, T-374A, Gly82Ser, G1704A) of *AGER* and its circulating forms with CAD via a meta-analysis of individual participant data from qualified case-controls studies, while addressing between-study heterogeneity and publication bias. Selection of these four polymorphisms is relatively straightforward if three or more unduplicated studies are available for a certain polymorphism.

## Methods

Meta-analysis of observational studies poses particular challenges due to the inherent biases and differences in study designs. In this context, we carried out this meta-analysis in accordance with the Preferred Reporting Items for Systematic Reviews and Meta-analyses (PRISMA) guideline [9] (see Supplementary Table S1).

### Search Strategy

A literature search was conducted of PubMed, EMBASE, Wanfang (http://www.wanfangdata.com.cn) and China National Knowledge Infrastructure (CNKI, http://www.cnki.net) databases covering the period from the earliest possible year to March 15, 2013. The following subject terms were used in the search: “advanced glycation end products”, “RAGE”, “AGER”, “coronary heart disease”, “coronary syndrome” or “isch[a]emic heart disease” or “vascular disease” or “myocardial infarction” or “atherosclerosis” or “arteriosclerosis” or “coronary stenosis” or “coronary artery disease” or “coronary disease” or “CAD” or “CHD” or “ACS”, combined with “gene” or “allele” or “genotype” or “polymorphism” or “variant” or “mutation”. The research was also supplemented by reviews of reference lists, hand-searching of relevant journals, and correspondence with authors. Search results were limited to studies on a case-control design and articles published in English or Chinese language.

### Study Selection

Two investigators (F.P. and W.N.) independently obtained the full texts of articles deemed as potentially eligible according to the titles and abstracts. If necessary, we emailed the contributing authors to avoid double counting of participants recruited in more than one study by the same group. Where more than one publication of the same study population existed, we abstracted data from the most recent or most complete publication.

### Inclusion/Exclusion Criteria

Our analyses were restricted to studies that fulfilled the following inclusion criteria (all must be satisfied): (1) clinical endpoint (dependent variable): CAD or myocardial infarction (MI); (2) study design: either retrospective or nested case-control; (3) independent parameters: either genotypes/alleles of at least one examined polymorphism or circulating sRAGE or endogenous secretory RAGE (esRAGE). Studies were excluded (one was sufficient) if they investigated the progression, severity, phenotype modification, response to treatment or survival, as well as if they were meeting abstracts, case reports/series, editorials, review articles, or non-English and non-Chinese articles.

### Data Extraction

Data were extracted from qualified articles independently by two investigators (F.P. and W.N.) according to a standardized Excel template (Microsoft Corp, Redmond, WA). Quality assessment was performed in duplicate with κ agreement rate of 0.98. Discrepancies were adjudicated by discussion and a consensus was reached. When three or more studies investigated the same polymorphism in *AGER* gene, published data were synthesized accordingly.

Collected data included the first author, publication year, ethnicity, CAD subtype (CAD or MI), study design, case-control status, genotypes/alleles of examined polymorphisms, circulating sRAGE and/or esRAGE levels, and the demographic records (if available), such as age, gender, matched information, percentage of diabetes and renal disease, body mass index, smoking, systolic and diastolic blood pressure.

### Statistical Analysis

To maximize power to detect a true association, only allelic and dominant models were adopted to estimate risk effects of *AGER* genetic polymorphisms on CAD. The random-effects model using the DerSimonian & Laird method was employed to calculate weighted odds ratio (OR) and the corresponding 95% confidence interval (95% CI). Comparisons of circulating sRAGE and esRAGE levels between patients and controls were expressed as weighted mean difference (WMD) with 95% CI.

Between-study heterogeneity was assessed by χ^2^ test, and was quantified using the *I* ^2^ statistic (ranging from 0 to 100%), which is defined as the percentage of the observed between-study variability that is due to heterogeneity rather than chance. Cumulative analyses were performed for all polymorphisms according to the ascending date of publication in order to identify the impact of the first-published article on subsequent publications, and the evolution of the pooled estimates over time.

Predefined subgroup analyses were performed a priori according to the CAD endpoints (CAD and MI), descents of study population (Caucasian, East Asian, Middle Eastern, African), study design (retrospective and prospective), matched information on age and/or gender, and total sample sizes (<300 subjects: small study and ≥300 subjects: large study). Meta-regression analyses were performed to evaluate the extent to which different study-level variables, including age, gender, body mass index, smoking, systolic and diastolic blood pressure, explained the heterogeneity of pooled risk estimates of *AGER* genetic polymorphisms examined or circulating sRAGE and esRAGE levels on CAD.

Publication bias was assessed by the Egger’s test and the trim-and-fill method. The latter was to estimate the number and outcomes of theoretically missing studies resulting from publication bias. P<0.05 was considered statistical significance, with the exception of *I* ^2^ and Egger’s statistics, for which a significance level was set at P<0.1 [10]. All statistical analyses were carried out with STATA software (StataCorp, TX, version 11.2 for Windows).

## Results

### Eligible Studies

**Figure 1:**
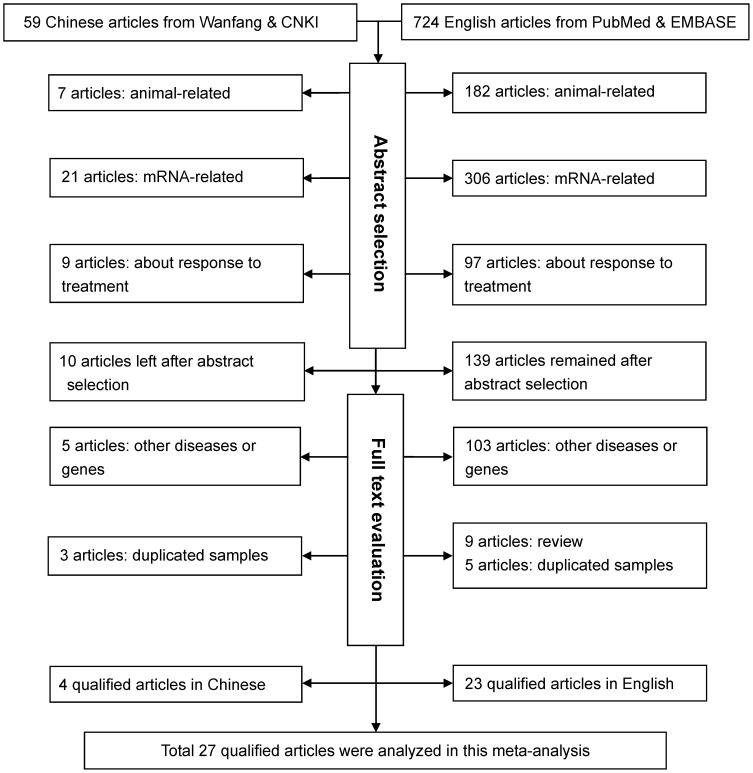
Flow diagram of search strategy and study selection.

Characteristics of all qualified studies in this meta-analysis are summarized in Table 1 and Table 2. The initial search yielded 783 potentially relevant articles. Applying our inclusion/exclusion criteria left 27 qualified articles [3], [5], [8], [11]–[34]. A flow diagram schematized the process of excluding articles with specific reasons (Figure 1). These 27 articles were published between 2004 and 2012, with 4 articles written in Chinese [22], [27], [30], [31] and the others in English. One article was grouped by ethnicity [13] and hypertension [24] respectively, three by CAD subtypes (CAD, MI, CAD with and without restenosis) [14], [17], [25], and five by diabetes mellitus [5], [8], [19], [20], [23]. These independent subgroups were treated separately, and accordingly there were 39 groups in final analyses.

**Table 1:** Characteristics of qualified studies.

Author (year)	Ethnicity	Matched	Disease	Diabetes (%)	Renal disease (%)	Study design
Kirbis (2004)	Caucasian	NA	CAD	100.00	NA	Retrospective
Falcone (2004)	Caucasian	NA	CAD	0.00	0.00	Retrospective
dos Santos (2005) (Caucasian)	Caucasian	NA	CAD	100.00	97.20	Retrospective
dos Santos (2005) (African)	African	NA	CAD	100.00	97.20	Retrospective
Falcone (2005)	Caucasian	NA	CAD	0.00	0.00	Retrospective
Hofmann (2005) (MI)	Caucasian	NA	MI	24.00	NA	Prospective
Hofmann (2005) (CAD-diabetes)	Caucasian	NA	CAD	100.00	NA	Prospective
Hofmann (2005) (CAD+diabetes)	Caucasian	NA	CAD	0.00	NA	Prospective
Zee (2006)	Caucasian	NA	MI	8.90	NA	Prospective
Yoon (2007)	Asian	NA	CAD	26.00	NA	Retrospective
Lu (2008) (−restenosis)	Asian	NA	CAD	100.00	0.00	Prospective
Lu (2008) (+restenosis)	Asian	NA	CAD	100.00	0.00	Prospective
Mulder (2008)	Caucasian	age, gender	CAD	19.00	0.00	Retrospective
Kucukhuseyin (2009) (+diabetes)	Middle Eastern	NA	CAD	100.00	NA	Retrospective
Kucukhuseyin (2009) (−diabetes)	Middle Eastern	NA	CAD	0.00	NA	Retrospective
Lu (2009) (−diabetes)	Asian	NA	CAD	0.00	NA	Retrospective
Lu (2009) (+diabetes)	Asian	NA	CAD	100.00	NA	Retrospective
Mahajan (2009)	Asian	NA	CAD	0.00	0.00	Retrospective
Peng (2009) (+diabetes)	Asian	NA	CAD	100.00	0.00	Retrospective
Peng (2009) (−diabetes)	Asian	NA	CAD	100.00	0.00	Retrospective
Yan (2009) (+diabetes)	Asian	NA	CAD	100.00	NA	Retrospective
Yan (2009) (−diabetes)	Asian	NA	CAD	0.00	NA	Retrospective
Pu (2009)	Asian	NA	CAD	100.00	NA	Retrospective
Gao (2010) (−hypertension)	Asian	age, gender	CAD	0.00	0.00	Retrospective
Gao (2010) (+hypertension)	Asian	age, gender	CAD	0.00	0.00	Retrospective
McNair (2010)	Caucasian	age, gender	MI	0.00	NA	Prospective
McNair (2010) (+restenosis)	Caucasian	NA	MI	0.00	NA	Retrospective
McNair (2010) (−restenosis)	Caucasian	NA	MI	0.00	NA	Retrospective
Xie (2010)	Asian	NA	CAD	13.39	0.00	Retrospective
Hou (2011)	Asian	NA	CAD	0.00	NA	Retrospective
Boiocchi (2011)	Caucasian	age, gender	MI	27.00	NA	Retrospective
Cai (2011) (CAD)	Asian	NA	CAD	45.50	0.00	Retrospective
Cai (2011) (MI)	Asian	NA	MI	46.20	0.00	Retrospective
Park (2011) (+diabetes)	Asian	age, gender	MI	100.00	0.00	Retrospective
Park (2011) (−diabetes)	Asian	age, gender	MI	0.00	0.00	Retrospective
Peng (2011)	Asian	NA	CAD	100.00	0.00	Retrospective
Lu (2011)	Asian	NA	CAD	0.00	NA	Retrospective
Aydogan (2012)	Middle Eastern	NA	CAD	0.00	NA	Retrospective
Selejan (2012)	Caucasian	NA	MI	35.00	0.00	Retrospective

*Abbreviations*: NA, not available; CAD, coronary artery disease; MI, myocardial infarction.

**Table 2:** Characteristics of study populations in qualified studies.

Author (year)	Age, yr	Males, %	BMI, kg/m^2^	Smoking	SBP, mmHg	DBP, mmHg	sRAGE, pg/ml	esRAGE, pg/ml
Kirbis (2004)	59.3/66.9	64.9/43.2	28.7/27.8	44/14.1	146/145	83/85	NA	NA
Falcone (2004)	61.8/59.6	79.4/77.4	26.1/24.9	73.7/45.2	NA	NA	NA	NA
dos Santos (2005) (Caucasian)	61.8/62.4	55.93/40.23	27.9/28.3	NA	NA	NA	NA	NA
dos Santos (2005) (African)	59.5/58.7	41.33/25	28.7/28.6	NA	NA	NA	NA	NA
Falcone (2005)	64.1/63.2	NA	25.7/25.6	49.09/31.1	NA	NA	966/1335	NA
Hofmann (2005) (MI)	NA	NA	NA	NA	NA	NA	NA	NA
Hofmann (2005) (CAD-diabetes)	NA	NA	NA	NA	NA	NA	NA	NA
Hofmann (2005) (CAD+diabetes)	NA	NA	NA	NA	NA	NA	NA	NA
Zee (2006)	NA	NA	NA	NA	NA	NA	NA	NA
Yoon (2007)	55.73/53.18	NA	25.01/23.41	84.9/77.3	119.86/114.16	77.13/75.84	NA	NA
Lu (2008) (−restenosis)	67/61	NA	NA	29.6/	140/	83/	NA	220/480
Lu (2008) (+restenosis)	65/61	NA	NA	33.7/14.7	137/137	81/80	NA	160/300
Mulder (2008)	64.7/63.4	78/72	27.7/25.5	68/72	NA	NA	1373/1299	NA
Kucukhuseyin (2009) (+diabetes)	61.42/57.96	42/49	27.48/25.52	40.7/49	135.59/123.6	85.13/76.2	NA	NA
Kucukhuseyin (2009) (−diabetes)	58.42/57.96	21/49	25.81/25.81	77.1/49	127.3/123.6	79.8/123.6	NA	NA
Lu (2009) (−diabetes)	64.8/56.3	74.16/49.57	NA	30.3/16.8	130/127	79/79	NA	NA
Lu (2009) (+diabetes)	66.5/62.8	68.90/36.09	NA	26.9/12.3	137/135	79/79	NA	NA
Mahajan (2009)	44.4/41.6	81/67.5	22.68/22.55	47/38	125.47/121.95	81.65/77.5	892.39/1611.9	NA
Peng (2009) (+diabetes)	64/63	63/58	25.41/25.65	41.6/25.1	136/135	NA	NA	270/290
Peng (2009) (−diabetes)	64/63	63/58	25.41/25.65	41.6/25.1	136/135	NA	NA	270/290
Yan (2009) (+diabetes)	66.2/62.3	70.19/45	NA	30.5/20	137/136	80/81	673.6/473.6	230/290
Yan (2009) (−diabetes)	64.1/58.6	72.48/53.03	NA	35.6/13.6	128/123	79/75	669.8/759.6	390/470
Pu (2009)	67.06/63.41	68.91/36.09	NA	26.89/12.25	137.19/135.22	79.16/79.25	NA	220/310
Gao (2010) (−hypertension)	60.8/61	74.3/42.4	NA	NA	120/118.3	72.7/71.7	NA	NA
Gao (2010) (+hypertension)	63.5/60.6	69/49.5	NA	NA	146.2/156	82.4/88.2	NA	NA
McNair (2010)	NA	NA	NA	NA	NA	NA	910.5/1302.5	NA
McNair (2010) (+restenosis)	61.5/60	NA	25/25	NA	148/125	74/78	610.6/1287	NA
McNair (2010) (−restenosis)	66.1/60	NA	29/25	NA	153/125	70/78	1143.8/1287	NA
Xie (2010)	NA	NA	NA	NA	NA	NA	NA	NA
Hou (2011)	57.7/58.2	78.15/77.34	NA	61.34/33.2	NA	NA	NA	NA
Boiocchi (2011)	59/62	83/64	26.1/25.6	NA	NA	NA	NA	NA
Cai (2011) (CAD)	65.5/61	58.3/53.8	25.4/25.5	25.1/17.1	132.5/130.2	78.9/78.6	691.53/652.55	NA
Cai (2011) (MI)	65.9/61	75.7/53.8	25/25.5	43.1/17.1	128.9/130.2	76.3/78.6	724.01/652.55	NA
Park (2011) (+diabetes)	64.2/62.2	50/50	23.8/24.9	44.4/27.8	NA	NA	610/450	NA
Park (2011) (−diabetes)	64.2/62.2	50/50	23.8/24.9	44.4/27.8	NA	NA	600/370	NA
Peng (2011)	68/64	63/53	25/25.7	24.9/25.9	139/137	78/79	NA	260/310
Lu (2011)	63.7/61.8	65.18/58.03	24.3/24.2	48.1/38.4	NA	NA	NA	NA
Aydogan (2012)	60.02/58.1	NA	25.92/25.52	NA	131.01/122.34	82.08/75.74	NA	NA
Selejan (2012)	NA	NA	NA	NA	NA	NA	122.15/125.68	NA

*Abbreviations*: NA, not available; BMI, body mass index; SBP, systolic blood pressure; DBP, diastolic blood pressure.

### Study Characteristics

Out of 39 qualified groups, 21 included East Asian, 14 included Caucasian, 4 included Middle Eastern, and 1 included African. Seven groups were reportedly matched in age and/or gender between patients and controls. There were 32 groups designed retrospectively and 7 groups prospectively. The patients of 10 groups were clinically diagnosed as MI.

Four polymorphisms of *AGER* gene were examined, including T-429C (rs1800625 in the promoter), T-374A (rs1800624 in the promoter), Gly82Ser (rs2070600 in exon 3) and G1704A (rs184003 in intron 7), and their results were extracted and synthesized in this meta-analysis. In detail, there were 10 (patients/controls: 1945/2013), 14 (2796/2209), 14 (2145/4966), and 3 (1075/1173) groups evaluating the association of these four polymorphisms with CAD. With regard to circulating sRAGE and esRAGE, there were respectively 13 (patients/controls: 1578/1275) and 8 (1752/1860) groups.

### Overall Analyses of AGER Genetic Polymorphisms

**Figure 2:**
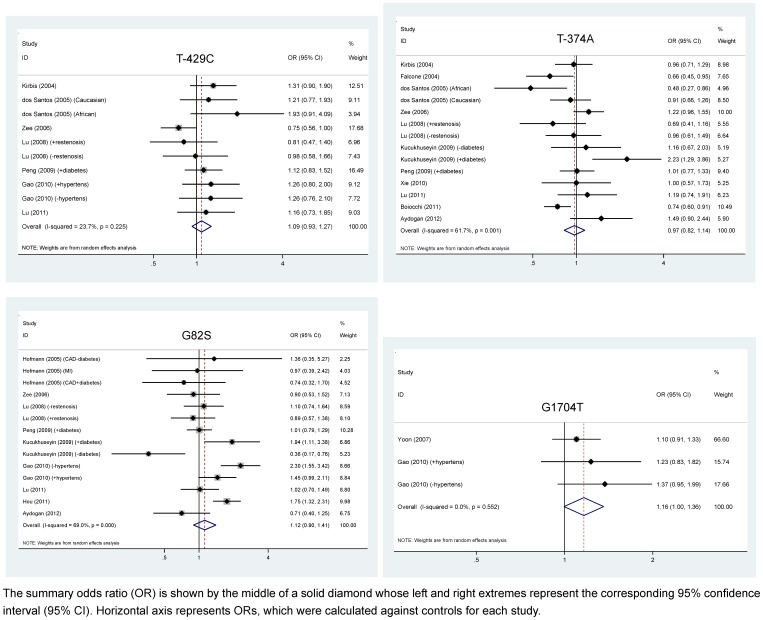
Overall estimates of *AGER* gene four polymorphisms examined for CAD under allelic model. The summary odds ratio (OR) is shown by the middle of a solid diamond whose left and right extremes represent the corresponding 95% confidence interval (95% CI). Horizontal axis represents ORs, which were calculated against controls for each study.

**Figure 3:**
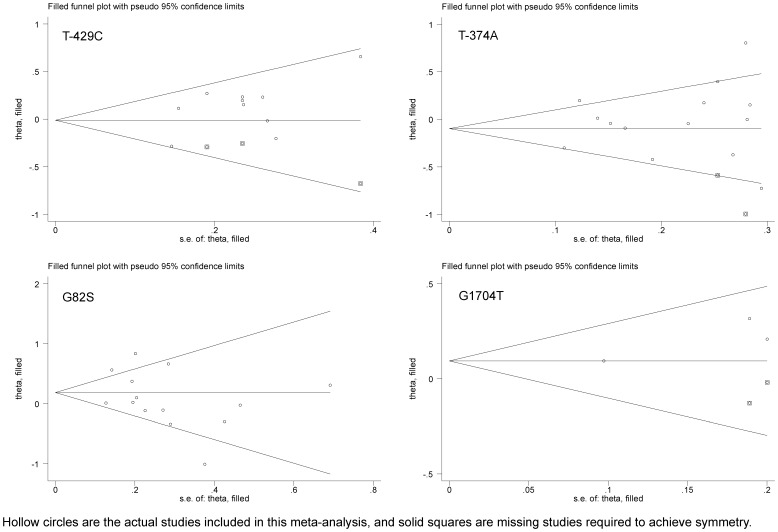
Trim-and-fill funnel plots for studies evaluating the effect of *AGER* gene four examined polymorphisms on CAD. Hollow circles are the actual studies included in this meta-analysis, and solid squares are missing studies required to achieve symmetry.

The fact that only three groups were available for G1704A precluded further subgroup analyses. Pooling all qualified groups detected no statistical significance for *AGER* gene four polymorphisms in association with CAD under allelic and dominant models (Figure 2 and Tables 3–5). Further restricting groups to CAD patients with diabetes mellitus found that deviations of risk estimates from the unity were stronger than the overall estimates, except for Gly82Ser under allelic model. For example, under dominant model, having -429C allele increased the odds of developing CAD in diabetic patients by 1.22-fold (95% CI: 0.99–1.51) compared with that of overall estimate of 1.15-fold (95% CI: 0.97–1.36). Contrastingly, the risk magnitude was alleviated for T-429C, T-374A, and Gly82Ser in CAD patients without diabetes mellitus. Moreover, in CAD patients without renal disease, deviations of risk estimates from the unity was enhanced, albeit non-significant, than the overall estimates, especially for Gly82Ser (allelic model: OR = 1.26; 95% CI: 0.92–1.74 and dominant model: OR = 1.28; 95% CI: 0.83–1.96). Significant heterogeneity was observed for T-374A (allelic model only) and Gly82Ser. There was high probability of publication bias for T-374A and Gly82Ser in CAD patients without diabetes mellitus. Further evidence of selective publication indicated that there were respectively three, two, and two missing groups required to make the funnel plot symmetrical for T-429C, T-374A and G1704A (Figure 3).

**Table 3:** Overall and subgroup analyses of *AGER* gene T-429C polymorphism with the risk of developing CAD, and exploration of between-study heterogeneity and publication bias.

Groups and subgroups	Studies (cases/controls), n (n/n)	Allele model		Dominant model	
		OR; 95% CI; *P*	*I* ^2^ (*P*_χ2_); *P*_Egger_	OR; 95% CI; *P*	*I* ^2^ (*P*_χ2_); *P*_Egger_
**Total studies**	10 (1945/2013)	1.09; 0.93–1.38; 0.301	23.7% (0.225); 0.118	1.15; 0.97–1.36; 0.111	18.0% (0.277); 0.097
**Total studies in DM**	6 (1004/1191)	1.15; 0.96–1.38; 0.118	0.0% (0.515); 0.792	1.22; 0.99–1.51; 0.06	6.7% (0.373); 0.554
**Total studies in non-DM**	4 (941/822)	1.04; 0.77–1.39; 0.815	49.3% (0.115); 0.013	1.07; 0.81–1.4; 0.644	30.8% (0.228); 0.026
**Total studies in non-RD**	5 (914/1094)	1.1; 0.9–1.33; 0.349	0.0% (0.74); 0.61	1.17; 0.94–1.45; 0.159	0.0% (0.787); 0.553
**CAD endpoint**					
CAD	9 (1604/1672)	1.17; 1.01–1.36; 0.073	0.0% (0.814); 0.642	1.24; 1.05–1.46; 0.012	0.0% (0.693); 0.411
MI	1 (341/341)	0.75; 0.56–1.0; 0.05	NA	0.8; 0.58–1.11; 0.181	NA
**Descent of populations**					
Caucasian	3 (686/751)	1.04; 0.71–1.52; 0.855	69.1% (0.039); 0.356	1.06; 0.75–1.5; 0.742	51.9% (0.125); 0.335
East Asian	6 (1184/1206)	1.11; 0.93–1.33; 0.266	0.0% (0.841); 0.901	1.17; 0.96–1.42; 0.13	0.0% (0.886); 0.465
African	1 (75/56)	1.93; 0.91–4.09; 0.088	NA	2.77; 1.18–6.53; 0.02	NA
**Study design**					
Retrospective	7 (1390/1315)	1.23; 1.05–1.45; 0.011	0.0% (0.928); 0.046	1.29; 1.08–1.55; 0.013	0.0% (0.737); 0.035
Prospective	3 (555/698)	0.82; 0.64–1.01; 0.052	0.0% (0.677); 0.403	0.85; 0.66–1.12; 0.232	0.0% (0.678); 0.531
**Age and/or gender**					
Matched	2 (330/369)	1.26; 0.9–1.78; 0.184	0.0% (0.944); 1.0	1.33; 0.92–1.91; 0.13	0.0% (0.933); 1.0
Unclear	8 (1615/1644)	1.06; 0.88–1.28; 0.538	27.2% (0.202); 0.18	1.12; 0.92–1.91; 0.275	30.6% (0.183); 0.184
**Total samples**					
<300 subjects	8 (1234/1304)	1.19; 1.0–1.42; 0.045	71.6% (0.06); 0.83	0.97; 0.66–1.43; 0.884	62.9% (0.101); 0.425
≥300 subjects	2 (711/709)	0.91; 0.62–1.35; 0.654	0.0% (0.741); 0.779	1.25; 1.04–1.52; 0.021	0.0% (0.598); 1.0

*Abbreviations:* DM, diabetes mellitus; RD, renal disease; CAD, coronary artery disease; MI, myocardial infarction; OR, odds ratio; 95% CI: 95% confidence interval; NA, not available.

**Table 4:** Overall and subgroup analyses of *AGER* gene T-374A polymorphism with the risk of developing CAD, and exploration of between-study heterogeneity and publication bias.

Groups and subgroups	Studies (cases/controls), n (n/n)	Allele model		Dominant model	
		OR; 95% CI; *P*	*I* ^2^ (*P*_χ2_); *P*_Egger_	OR; 95% CI; *P*	*I* ^2^ (*P*_χ2_); *P*_Egger_
**Total studies**	14 (2796/2209)	0.97; 0.82–1.14; 0.713	61.7% (0.001); 0.567	0.97; 0.82–1.13; 0.658	31.7% (0.122); 0.876
**Total studies in DM**	7 (1058/1246)	0.94; 0.74–1.21; 0.644	63.2% (0.012); 0.822	0.91; 0.72–1.17; 0.465	41.1% (0.117); 0.717
**Total studies in non-DM**	7 (1738/963)	1.0; 0.78–1.27; 0.987	66.0% (0.007); 0.417	1.02; 0.82–1.26; 0.89	28.2% (0.213); 0.838
**Total studies in non-RD**	5 (886/899)	0.87; 0.72–1.05; 0.137	11.3% (0.341); 0.57	0.91; 0.73–1.12; 0.363	0.0% (0.91); 0.577
**CAD endpoint**					
CAD	12 (1764/1634)	0.98; 0.81–1.18; 0.801	55.2% (0.011); 0.71	0.96; 0.81–1.13; 0.595	15.3% (0.295); 0.972
MI	2 (1032/575)	0.95; 0.58–1.54; 0.821	89.1% (0.002); 1.0	0.99; 0.58–1.69; 0.976	83.2% (0.015); 1.0
**Descent of populations**					
Caucasian	5 (1552/1069)	0.89; 0.71–1.1; 0.279	67.1% (0.016); 0.817	0.95; 0.75–1.2; 0.662	46.2% (0.115); 0.516
East Asian	5 (976/926)	0.98; 0.81–1.17; 0.806	0.0% (0.649); 0.627	0.99; 0.8–1.22; 0.893	0.0% (0.872); 0.86
Middle Eastern	3 (193/158)	1.57; 1.09–2.24; 0.014	26.8% (0.255); 0.969	1.37; 0.88–2.13; 0.168	0.0% (0.659); 0.596
African	1 (75/56)	0.48; 0.27–0.86; 0.013	NA	0.36; 0.18–0.74; 0.006	NA
**Study design**					
Retrospective	11 (2241/1511)	0.97; 0.8–1.18; 0.751	64.3% (0.002); 0.198	0.93; 0.77–1.11; 0.425	31.3% (0.149); 0.573
Prospective	3 (555/698)	0.99; 0.72–1.37; 0.956	50.9% (0.131); 0.176	1.12; 0.86–1.46; 0.408	11.1% (0.325); 0.095
**Age and/or gender**					
Matched	1 (691/234)	0.74; 0.6–0.92; 0.005	NA	0.76; 0.55–1.03; 0.079	NA
Unclear	13 (2105/1975)	1.01; 0.85–1.19; 0.697	56.1% (0.007); 0.805	1.0; 0.85–1.18; 0.991	25.6% (0.185); 0.493
**Total samples**					
<300 subjects	11 (1394/1264)	0.97; 0.79–1.2; 0.808	59.0% (0.007); 0.579	0.96; 0.79–1.17; 0.659	23.0% (0.225); 0.992
≥300 subjects	3 (1402/945)	0.96; 0.71–1.31; 0.811	79.0% (0.008); 0.552	0.98; 0.72–1.33; 0.892	66.8% (0.049); 0.331

*Abbreviations:* DM, diabetes mellitus; RD, renal disease; CAD, coronary artery disease; MI, myocardial infarction; OR, odds ratio; 95% CI: 95% confidence interval; NA, not available.

**Table 5:** Overall and subgroup analyses of *AGER* gene Gly82Ser and G1704A polymorphisms with the risk of developing CAD, and exploration of between-study heterogeneity and publication bias.

Groups and subgroups	Studies (cases/controls), n (n/n)	Allele model		Dominant model	
		OR; 95% CI; *P*	*I* ^2^ (*P*_χ2_); *P*_Egger_	OR; 95% CI; *P*	*I* ^2^ (*P*_χ2_); *P*_Egger_
**Gly82Ser polymorphism**					
**Total studies**	14 (2145/4966)	1.12; 0.9–1.41; 0.316	69.0% (<0.001); 0.259	1.12; 0.82–1.52; 0.477	75.8% (<0.001); 0.707
**Total studies in DM**	5 (678/900)	1.11; 0.88–1.39; 0.381	26.0% (0.248); 0.415	1.2; 0.77–1.87; 0.423	66.8% (0.017); 0.235
**Total studies in non–DM**	9 (1467/4066)	1.08; 0.77–1.5; 0.673	76.6% (<0.001); 0.028	1.04; 0.68–1.59; 0.851	79.6% (<0.001); 0.023
**Total studies in non–RD**	5 (914/1097)	1.26; 0.92–1.74; 0.148	73.8% (0.004); 0.562	1.28; 0.83–1.96; 0.262	80.1% (<0.001); 0.484
**CAD endpoint**					
CAD	12 (1739/3058)	1.15; 0.9–1.47; 0.278	72.8% (<0.001); 0.348	1.15; 0.81–1.62; 0.435	79.1% (<0.001); 0.789
MI	2 (406/1908)	0.91; 0.58–1.45; 0.702	0.0% (0.881); 1.0	0.91; 57–1.46; 0.696	0.0% (0.878); 1.0
**Descent of populations**					
Caucasian	4 (538/3408)	0.9; 0.61–1.33; 0.601	0.0% (0.898); 0.48	0.9; 0.61–1.33; 0.592	0.0% (0.893); 0.473
East Asian	7 (1407/1396)	1.29; 1.01–1.66; 0.046	72.5% (0.001); 0.968	1.32; 0.95–1.83; 0.099	75.6% (<0.001); 0.404
Middle Eastern	3 (200/162)	0.81; 0.32–2.06; 0.662	85.5% (0.003); 0.425	0.83; 0.18–3.89; 0.807	91.3% (<0.001); 0.872
**Study design**					
Retrospective	8 (1393/1201)	1.21; 0.88–1.67; 0.239	80.7% (<0.001); 0.496	1.24; 0.78–1.97; 0.372	85.7% (<0.001); 0.951
Prospective	6 (752/3765)	0.96; 0.76–1.22; 0.75	0.0% (0.939); 0.911	0.93; 0.72–1.21; 0.605	0.0% (0.958); 0.81
**Age and/or gender**					
Matched	2 (330/370)	1.82; 1.16–2.87; 0.01	63.9% (0.096); 1.0	2.08; 1.23–3.51; 0.006	63.4% (0.098); 1.0
Unclear	12 (1815/4596)	1.02; 0.81–1.28; 0.864	61.8% (0.002); 0.225	0.99; 0.73–1.34; 0.926	68.5% (<0.001); 0.84
**Total samples**					
<300 subjects	12 (1434/4255)	0.99; 0.79–1.24; 0.909	0.0% (0.693); 0.105	0.88; 0.68–1.15; 0.353	0.0% (0.972); 0.275
≥300 subjects	2 (711/711)	1.15; 0.88–1.51; 0.309	70.9% (<0.001); 1.0	1.17; 0.81–1.68; 0.414	77.0% (<0.001); 1.0
**G1704A polymorphism**					
**Total studies**	3 (1075/1173)	1.16; 1.0–1.36; 0.057	0.0% (0.552); 0.28	1.1; 0.92–1.31; 0.307	0.0% (0.907); 0.41

*Abbreviations:* DM, diabetes mellitus; RD, renal disease; CAD, coronary artery disease; MI, myocardial infarction; OR, odds ratio; 95% CI: 95% confidence interval; NA, not available.

### Overall Analyses of Circulating RAGE Forms

Circulating sRAGE levels were lower in CAD patients than in controls with the difference being non-significant (Table 6). However, this reduction was totally and significantly reversed in CAD patients with diabetes mellitus (WMD: 185.71 pg/ml; 95% CI: 106.82 to 264.61 pg/ml), without evidence of heterogeneity or publication bias. Relative to controls, circulating esRAGE levels were consistently and significantly lower in CAD patients, as well as in subgroups with or without diabetes mellitus, and without renal disease. However, significant heterogeneity obsessed these comparisons, and the probability of publication bias was low.

**Table 6:** Overall and subgroup analyses of circulating sRAGE and esRAGE levels with CAD, and exploration of between-study heterogeneity and publication bias.

sRAGE and esRAGE levels	Studies (cases/controls),n (n/n)	WMD; 95% CI; P	*I* ^2^ (*P*_χ2_); *P*_Egger_
**sRAGE (pg/ml)**			
**Total studies**	13 (1578/1275)	−123.12; −294.63 to 48.39; 0.159	99.7% (<0.001); 0.664
**Total studies in DM**	3 (232/161)	185.71; 106.82 to 264.61; <0.001	0.0% (0.634); 1.0
**Total studies in non-DM**	11 (1400/1168)	−177.9; −363.33 to 7.54; 0.06	99.7% (<0.001); 0.861
**Total studies in non-RD**	9 (1250/1031)	−54.46; −216.05 to 107.13; 0.509	98.9% (<0.001); 0.966
**CAD endpoint**			
CAD	6 (1002/864)	−136.54; −370.88 to 97.8; 0.253	98.2% (<0.001); 0.261
MI	7 (576/411)	−110.61; −362.23 to 141.01; 0.389	99.8% (<0.001); 0.863
**Descent of populations**			
Caucasian	6 (493/467)	−257.72; −509.63 to −5.8; 0.045	99.9% (<0.001); 0.721
East Asian	7 (1085/808)	15.26; −104.78 to 135.29; 0.803	91.8% (<0.001); 0.512
**Study design**			
Retrospective	12 (1542/1245)	−100.25; −291.8 to 91.31; 0.305	99.7% (<0.001); 0.662
Prospective	1 (36/30)	−392.0; −471.43 to −366.57; <0.001	NA
**Age and/or gender**			
Matched	4 (153/117)	14.92; −381.82 to 411.66; 0.941	98.3% (<0.001); 0.079
Unclear	9 (1425/1158)	−182.65; −402.44 to 37.14; 0.103	99.8% (<0.001); 0.877
**Total samples**			
<300 subjects	3 (929/830)	−86.94; −411.07 to 237.19; 0.599	99.3% (<0.001); 0.857
≥300 subjects	10 (619/445)	−134.55; −347.88 to 78.78; 0.216	99.7% (<0.001); 0.136
**esRAGE (pg/ml)**			
**Total studies**	9 (1752/1860)	−84.27; −133.94 to −34.61; 0.001	96.4% (<0.001); 0.675
**Total studies in DM**	8 (1603/1728)	−84.6; −140.64 to −28.56; 0.003	96.8% (<0.001); 0.711
**Total studies in non-DM**	1 (149/132)	−80.0; −109.38 to −50.62; <0.001	NA
**Total studies in non-RD**	6 (1095/1346)	−117.82; −222.58 to −13.05; 0.028	98.2% (<0.001); 0.681
**CAD endpoint**			
CAD	8 (1698/1806)	−90.25; −142.34 to −38.17; 0.001	96.8% (<0.001); 0.764
MI	1 (54/54)	−20.0; −110.61 to 70.61; 0.665	NA
**Descent of populations**			
East Asian	9 (1752/1860)	−84.27; −133.94 to −34.61; 0.001	96.4% (<0.001); 0.675
**Study design**			
Retrospective	7 (1538/1503)	−52.12; −74.48 to −26.76; <0.001	79.1% (<0.001); 0.332
Prospective	2 (214/357)	−199.7; −317.3 to −82.1; 0.001	97.6% (<0.001); 1.0
**Age and/or gender**			
Matched	1 (54/54)	−20.0; −110.61 to 70.61; 0.665	NA
Unclear	8 (1698/1806)	−90.25; −142.34 to −38.17; 0.001	96.8% (<0.001); 0.764
**Total samples**			
<300 subjects	4 (1184/1237)	−45.62; −85.61 to −5.62; 0.025	88.4% (<0.001); 0.724
≥300 subjects	5 (568/623)	−116.06; −192.92 to −39.12; 0.003	96.8% (<0.001); 0.366

*Abbreviations*: WMD, weighted mean difference; 95% CI, 95% confidence interval; DM, diabetes mellitus; RD, renal disease; CAD, coronary artery disease; MI, myocardial infarction; NA, not available.

### Subgroup Analyses

As for T-429C, significance was reached in patients with CAD (compared with MI) under both models, in populations of African descent under dominant model, in studies under retrospective design and involving more than 300 subjects (Table 3). As for T-374A, significance was reached in populations of Middle Eastern (under allelic model) and African (under both models) descents, and in studies with age and/or gender-matched controls under allelic model (Table 4). As for Gly82Ser, significance was reached in populations of East Asian descent under both models, and in studies with age and/or gender-matched controls under dominant model (Table 5). As expected, heterogeneity was greatly improved in subgroups except for Gly82Ser in East Asians. Likewise, publication bias was also greatly improved in subgroups except for T-429C in retrospectively-designed studies.

Circulating sRAGE levels were significantly lower in CAD patients of Caucasian descent and in prospectively-designed studies than controls (Table 6). With regard to circulating esRAGE, significant lower levels were observed in studies under retrospective or prospective design, and in small or large studies than controls, especially for the prospective and large studies (Table 6).

### Cumulative and Met-regression Analyses

Regarding four examined polymorphisms of *AGER* and its circulating forms, cumulative risk estimates tended to be stable with accumulating data over time under both models (data not shown).

To explore the extent to which study-level variables explain heterogeneity among individual estimates, a set of meta-regression analyses were undertaken. Unfortunately, none of the confounders including age, gender, body mass index, smoking, systolic and diastolic blood pressure could explain large part of heterogeneity for all examined polymorphisms and circulating sRAGE and esRAGE levels (data not shown). Because meta-regression analyses involved studies of limited sample sizes, it might be underpowered to detect a small or moderate effect.

## Discussion

On the basis of 27 studies involving 7585 CAD patients and 9240 controls, we evaluated the association of *AGER* genetic polymorphisms and circulating sRAGE and esRAGE levels with the risk of developing CAD. The two noteworthy findings of this study were that (1) despite the overall null association, there was a contributory role of common variants in *AGER* gene to CAD in patients with diabetes mellitus or renal disease; (2) circulating esRAGE might be a powerful negative predictor for the development of CAD. Moreover, our findings demonstrated that the existence of diversity of ethnicity, study design, case-control matched information and sample size across studies might result to the presence of heterogeneity.

More recently, Wang and colleagues have synthesized data from 17 studies on *AGER* three genetic polymorphisms (T-429C, T-374A, Gly82Ser) and the risk of CAD, and they failed to observe any suggestive association [6], consistent with the pooled results of this meta-analysis. Extending beyond overall comparisons, we noticed that risk effects of *AGER* genetic variants on CAD were strikingly potentiated in patients with diabetes mellitus or renal disease. As indicated by clinical investigations, over-expression of *AGER* gene can enhance inflammatory reaction and matrix metalloprotease expression in plaque macrophages of diabetic patients [35]. Moreover, *AGER* expression was found to be closely associated with the worsening of chronic kidney disease [36]. Furthermore, circulating esRAGE levels were remarkably lower in type 2 diabetic subjects without chronic kidney disease than in nondiabetic controls, but gradually increased in accordance with progression of chronic kidney disease [37]. On the basis of previous work and the findings of this study, it is reasonable to hypothesize that diabetes mellitus and/or renal disease might precipitate the occurrence of CAD via the inheritance of genetic defects leading to the transcriptional activation of *AGER*.

To shed some light on this hypothesis, we further evaluated the relation between circulating RAGE forms, which can serve as RAGE blockers and might be applicable to human diseases, and CAD, and it is worth noting that circulating esRAGE might be a powerful negative predictor of CAD, even with the presence of diabetes mellitus or renal disease. Specifically, esRAGE is an alternative splicing product of *AGER* mRNA, constituting approximately 20% of sRAGE levels in humans [38]. There is growing evidence that circulating esRAGE was decreased in both types 1 and 2 diabetic patients, and its low levels were associated with the severity of cardiac dysfunction in patients with heart failure [2], [32], [39], [40], a finding which is also mirrored in this meta-analysis. In fact, therapies targeting RAGE have been undertaken in experimental models and are proven to be effective in reducing atherosclerosis in diabetic mice [41], [42]. Also, the soundness of our results on esRAGE was bolstered by further restricting subgroup analyses to the prospectively-designed and large studies, which are deemed to be less prone to selective publication and change results. This study, to our knowledge, represents the first meta-analysis to date evaluating the association of circulating sRAGE and esRAGE with CAD.

Heterogeneity in a meta-analysis is mostly produced by differences in study-level characteristics. Our subgroup analyses indicated that ethnicity, study design, matched information and sample size might be potential sources of heterogeneity. For example, risk estimate of 82Ser allele on CAD was 1.29 in East Asians, but was 0.9 in Caucasians. A possible explanation may be due to divergent genetic backgrounds or linkage patterns, and usually a variant is in close linkage with another nearby causal variant in one ethnic group but not in another [43], [44]. As a consequence, there is a need to construct a database of CAD-susceptibility genes or variants in each racial/ethnic group.

Meta-analysis is a powerful tool to summarize results of individual studies; however, it is important to recognize certain limitations. First, most qualified studies were retrospective in design, precluding further comments on causality. Second, albeit low probability of publication bias in this meta-analysis, potential selection bias cannot be ruled out, because we only retrieved articles published in English or Chinese. Third, heterogeneity persisted in some subgroups, limiting the interpretation of our pooled estimates. Fourth, as most studies in this meta-analysis enrolled subjects aged more than 50 years, large studies in a younger population of CAD patients are of special interest, because genetic factors may exert great contribution to those in whom CAD develops at a younger age and in the absence of strong environmental risk factors [45]. Fifth, we selected only four polymorphisms from *AGER* gene, and did not cover other CAD-susceptibility genes, such as angiotensin II receptor, type 1 [44] and matrix metalloproteinase family genes [46]. Therefore, the jury must refrain from drawing a final conclusion until large, well-designed, prospective studies confirm or refute our findings.

Taken together, our findings collectively demonstrated that association of *AGER* genetic polymorphisms with CAD was potentiated in patients with diabetes mellitus or renal disease. From a practical standpoint, circulating esRAGE might be a powerful negative predictor for the development of CAD. Nevertheless, we hope that this study will establish background data for further investigations into the mechanisms of *AGER* gene and relevant pathway genes in the development of CAD.

## Supporting Information

Table S1 **Checklist of items to include when reporting a systematic review or meta-analysis.**
(DOC)
